# Developmental Potential of Prepubertal Mouse Oocytes Is Compromised Due Mainly to Their Impaired Synthesis of Glutathione

**DOI:** 10.1371/journal.pone.0058018

**Published:** 2013-03-01

**Authors:** Guang-Zhong Jiao, Xin-Yan Cao, Wei Cui, Hua-Yu Lian, Yi-Long Miao, Xiu-Fen Wu, Dong Han, Jing-He Tan

**Affiliations:** College of Animal Science and Veterinary Medicine, Shandong Agricultural University, Tai-an City, People’s Republic of China; Institute of Zoology, Chinese Academy of Sciences, China

## Abstract

Although oocytes from prepubertal animals are found less competent than oocytes from adults, the underlying mechanisms are poorly understood. Using the mouse oocyte model, this paper has tested the hypothesis that the developmental potential of prepubertal oocytes is compromised due mainly to their impaired potential for glutathione synthesis. Oocytes from prepubertal and adult mice, primed with or without eCG, were matured in vitro and assessed for glutathione synthesis potential, oxidative stress, Ca^2+^ reserves, fertilization and in vitro development potential. In unprimed mice, abilities for glutathione synthesis, activation, male pronuclear formation, blastocyst formation, cortical granule migration and polyspermic block were all compromised significantly in prepubertal compared to adult oocytes. Cysteamine and cystine supplementation to maturation medium significantly promoted oocyte glutathione synthesis and blastocyst development but difference due to maternal age remained. Whereas reactive oxygen species (ROS) levels increased, Ca^2+^ storage decreased significantly in prepubertal oocytes. Levels of both catalytic and modifier subunits of the γ-glutamylcysteine ligase were significantly lower in prepubertal than in adult oocytes. Maternal eCG priming improved all the parameters and eliminated the age difference. Together, the results have confirmed our hypothesis by showing that prepubertal oocytes have a decreased ability to synthesize glutathione leading to an impaired potential to reduce ROS and to form male pronuclei and blastocysts. The resulting oxidative stress decreases the intracellular Ca^2+^ store resulting in impaired activation at fertilization, and damages the microfilament network, which affects cortical granule redistribution leading to polyspermy.

## Introduction

The production of viable embryos from oocytes obtained from prepubertal animals would decrease the generation interval and therefore lead to a significant increase in the annual genetic gain over conventional progeny testing programs [1]. Furthermore, abattoir derived prepubertal ovaries constitute an economical source of oocytes for embryo biotechnology. However, although oocytes obtained from prepubertal animals have produced viable offspring after in vitro maturation and fertilization [2], [3], [4], [5], the developmental competence of these embryos was lower than that of embryos produced from adult animals [2], [3], [6]–[13].

In vitro-matured oocytes of prepubertal bovine showed abnormal chromatin microtubule configurations and protein synthesis [9], [14], [15]. Increased polyspermy has been observed in lamb compared with ewe oocytes [12]. Furthermore, the number of oocytes that failed to show Ca^2+^ oscillations after fertilization was significantly higher in calf than in cow [9]. Polyspermy results from inefficient zona reaction and/or ooplasmic block to sperm penetration due to failed or incomplete exocytosis of cortical granules (CGs) from the oocyte [16]. However, while calf oocytes showed abnormal CGs distribution [9), CGs distribution in oocytes from prepubertal goat and sheep was normal following in vitro maturation [11], [17].

Glutathione functions in catalysis, metabolism, transport and reductive processes and an increased cellular level of glutathione has been found to protect cells against radiation, free radicals, reactive oxygen intermediates and other toxic compounds [18]. In reproduction, the intra-oocyte level of glutathione has been used as an index of ooplasmic maturation, with oocytes with higher glutathione contents showing better developmental potentials [19]–[22]. The intra-oocyte glutathione not only participates in sperm decondensation and male pronucleus (PN) formation [23]–[25], but also it was important for the development of parthenogenetic embryos [26], [27]. However, studies on the effect of glutathione synthesis on the developmental potential of prepubertal oocytes reported different results in different species [24], [27]–[29].

Cells depend on de novo synthesis to maintain intracellular glutathione. Glutathione is synthesized in two sequential ATP-dependent enzymatic reactions that are catalyzed by glutamate cysteine ligase (GCL) and glutathione synthetase (GSS), respectively. The rate-limiting step in synthesis of glutathione is catalyzed by GCL, a heterodimer composed of a catalytic (GCLc) subunit and a modifier (GCLm) subunit [30], [31]. De novo synthesis of glutathione can be regulated by transcriptional and posttranscriptional regulation of GCL, by the availability of cysteine and by the negative feedback of glutathione on GCL. Although effects of gonadotropins on GCL subunit expression in ovaries have been studied [32], [33], there is no report on the effect of sexual maturity on glutathione synthesis and GCL subunit expression. Furthermore, reports on GCL expression in the oocyte itself are few [34], [35].

It is known that peripheral CGs migration during oocyte maturation is driven by the microfilaments [36], and the microfilament network was one of the earliest targets of oxidative stress [37]. Reactive oxygen species (ROS) are known to significantly perturb Ca^2+^ homeostasis mainly through direct effects on the machinery involved in intracellular Ca^2+^ storage [38], [39]. Furthermore, the intracellular Ca^2+^ store was decreased in the in vitro-aged oocytes that may have been exposed to oxidative stress for a prolonged time [40]. Therefore, we hypothesized that the developmental potential of prepubertal oocytes might be compromised due mainly to their impaired function of the glutathione system. Thus, oocytes obtained from prepubertal animals might have decreased ability to synthesize glutathione leading to an impaired potential to reduce ROS and to form male PN. The resulting oxidative stress would (i) decrease the intracellular Ca^2+^ store resulting in impaired activation at fertilization, (ii) damage the microfilament network and affect CGs redistribution leading to polyspermy. The present study has tested and confirmed the hypothesis using the mouse oocyte model.

## Results

### Maternal Sexual Maturation and eCG Treatment Improved Fertilization Mainly by Promoting Oocyte Activation and Male PN Development

Oocytes were matured and inseminated in vitro. At 8 h after insemination, oocytes were observed for sperm penetration and PN formation. Oocytes not penetrated by sperm were arrested at the metaphase II (MII) stage. Sperm-penetrated oocytes were classified into monospermic and polyspermic. Monospermic oocytes were penetrated by a single spermatozoon with the formation of only the female PN or both the female and male PN following extrusion of the second polar body (P2). Polyspermic oocytes were penetrated by two or more spermatozoa with the formation of 1 to 3 PN after P2 extrusion. In addition, some of the oocytes were not activated at all in spite of sperm penetration. Percentages of sperm-penetrated oocytes did not defer between treatments (Table 1). In unstimulated mice, rates of polyspermy, particularly the percentages of oocytes that failed to form male PN or not activated at all after sperm penetration, were significantly higher in prepubertal than in adult oocytes. In eCG-primed mice, however, the difference became insignificant between prepubertal and adult oocytes.

**Table 1:** Effects of maternal puberty and eCG treatment on fertilization of mouse oocytes following in vitro maturation.

Mice	eCG	% (No.) of Penetrated oocytes	% (No.) of Monospermic oocytes		% (No.) of Polyspermic oocytes		% (No.) of MII+1SP oocytes
			Total	1PN+1SP	Total	1PN+2SP	
Prepub	−	71.9±3.3(52/73)^a^	59.7±1.4(31)^a^	32.4±3.9(10)^a^	17.4±0.9(9)^a^	33.3±0(3)^a^	22.8±2.1(12)^a^
Adult	−	72.6±6.8(46/62)^a^	81.8±2.7(38)^b^	10.5±0.3(4)^b^	12.7±1.9(6)^b^	11.1±11.1(1)^b^	5.6±2.8(2)^b^
Prepub	+	71.5±5.9(51/72)^a^	72.7±3.1(37)^b^	8.3±4.8(3)^b^	13.7±1.5(7)^b^	16.6±16.6(1)^b^	13.7±1.5(7)^b^
Adult	+	75.0±6.1(58/78)^a^	81.0±1.0(47)^b^	8.2±1.4(4)^b^	12.0±0.9(7)^b^	0.0±0.0(0)^b^	6.9±1.8(4)^b^

a–b: Values without a common letter in their superscripts differ in the same column (P<0.05).

To further confirm the lower ability of prepubertal oocytes to initiate activation, we compared activation between prepubertal and adult oocytes following a weak activating stimulus (10% ethanol for 10 min). Whereas 83.7±3.9% (68/81) of adult oocytes were activated, only 63.2±5.3% (60/94) of prepubertal oocytes were activated after ethanol stimulation.

### Maternal Sexual Maturation and eCG Treatment Improved CGs Migration of Oocytes

**Figure 1:**
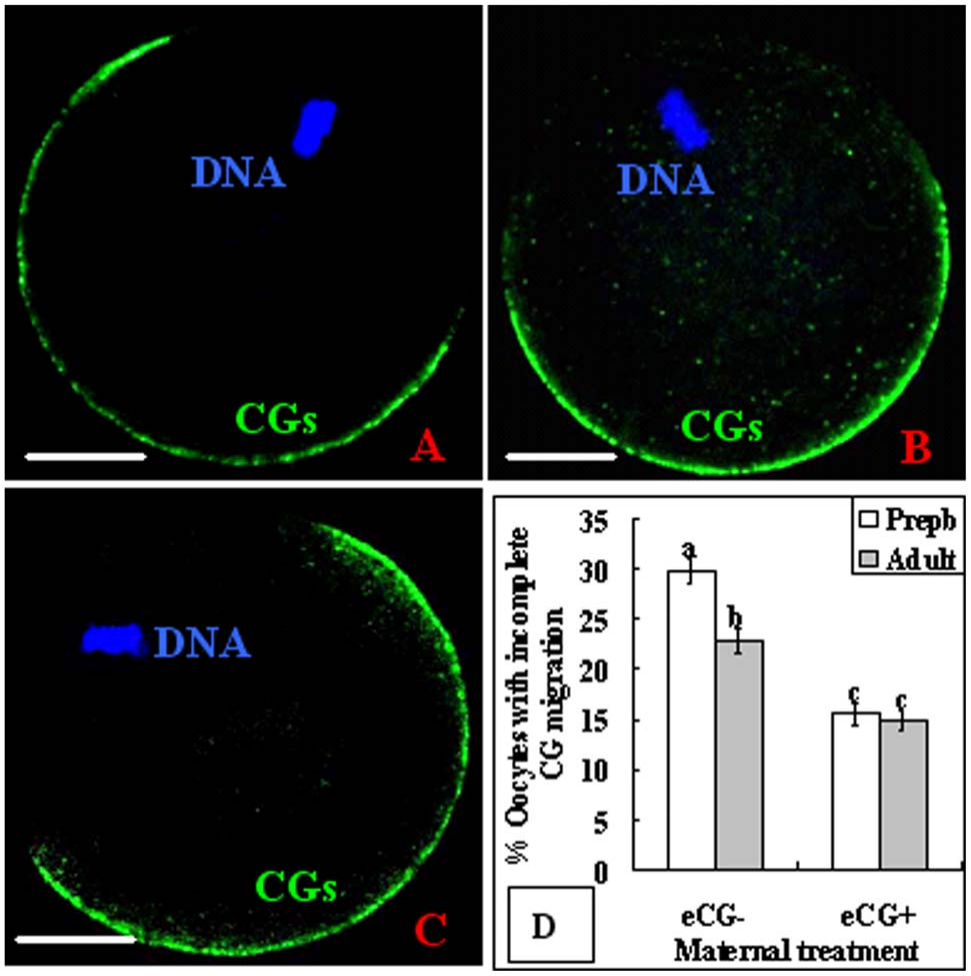
Effects of maternal puberty and eCG treatment on CGs distribution of mouse oocytes following in vitro maturation. A to C are confocal images. Image A is an oocyte with complete migration of CGs, whereas images B and C are oocytes with incomplete CGs migration. Oocytes were observed under a laser confocal microscope following immunostaining with Hoechst 33342 for DNA and with FITC-LCA for CGs. Scale bar is 20 µm. D is a graph showing percentages of oocytes with incomplete CGs migration in prepubertal (Prepb) or adult mice primed with (eCG+) or without eCG (eCG−). Each treatment was repeated 4–5 times and each replicate contained 25–35 oocytes. a–c: Values without a common letter above their bars differ (P<0.05).

In vitro matured oocytes were observed for CGs distribution at the end of maturation culture. Two types of CGs distribution were observed: (a) complete migration with all the CGs located in the egg cortex forming a monolayer beneath the plasma membrane (Fig. 1A) and (b) incomplete migration with some CGs having migrated into the cortex but still many left in the inner ooplasm (Fig. 1B and C). In unprimed mice, the percentage of oocytes with incomplete CGs migration was significantly higher in prepubertal than in adult mice (Fig. 1D). Treatment with eCG significantly decreased the number of oocytes with incomplete CGs migration in both prepubertal and adult mice and eliminated the age difference.

### Maternal Sexual Maturation and eCG Treatment Increased the Intra-oocyte Ca^2+^ Reserves

**Figure 2:**
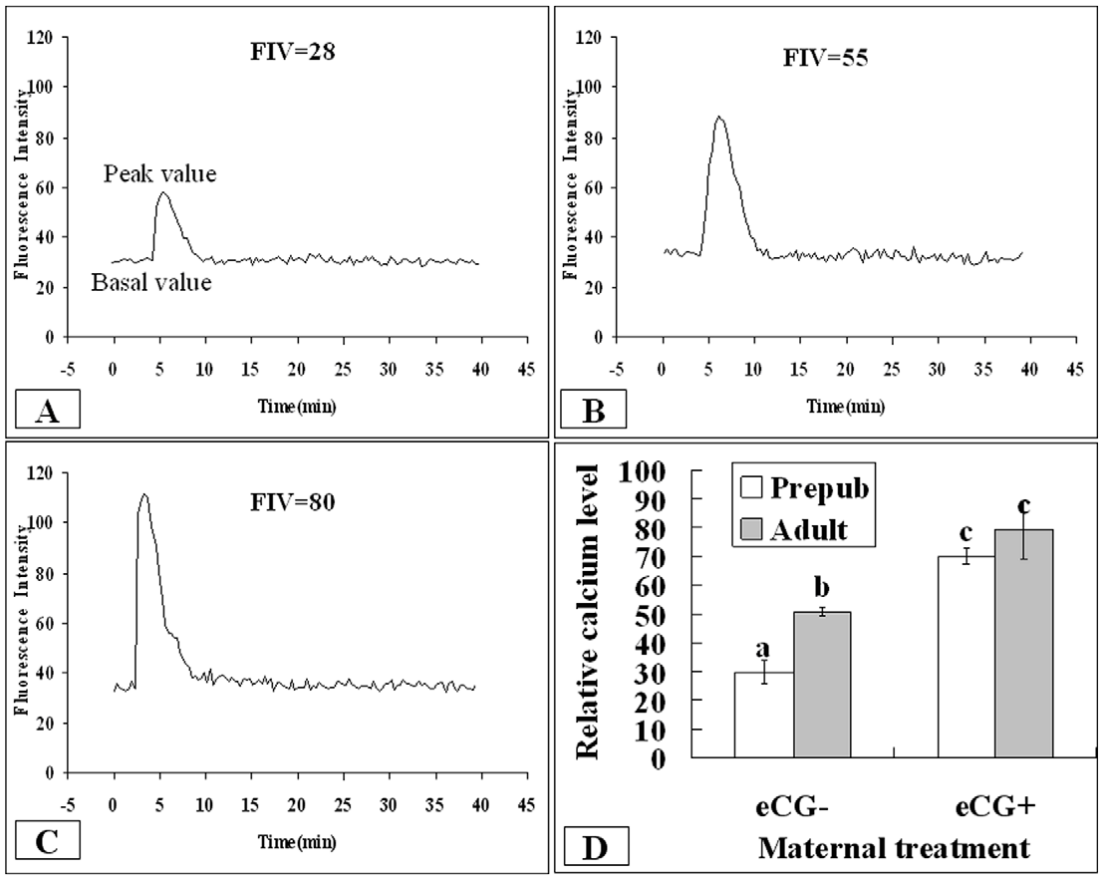
Effects of maternal puberty and eCG treatment on intra-oocyte Ca^2+^ reserves of mouse oocytes following in vitro maturation. The fluorescence intensity value (FIV) for each oocyte was calculated by subtracting the basal intensity value from the peak value. Panels A, B and C show three oocytes with a FIV of 30, 55 and 80, respectively, whereas panel D shows the relative levels of Ca^2+^ in oocytes from prepubertal (Prepb) or adult mice primed with (eCG+) or without eCG (eCG−). Each treatment was repeated three times with each replicate containing at least 20 oocytes. a–c: Values without a common letter above their bars differ (P<0.05).

In vitro matured oocytes were measured for Ca^2+^ reserves at the end of maturation culture. Intracellular Ca^2+^ levels of oocytes were determined according to their fluorescence intensity value (FIV), which was calculated by subtracting the basal intensity value from the peak intensity value. In unprimed mice, the relative calcium level was lower significantly in prepubertal than in adult mice (Fig. 2), indicating a marked decrease in Ca^2+^ reserves in the prepubertal oocytes. Difference due to maternal age became insignificant after eCG priming.

### Maternal Sexual Maturation and eCG Treatment Decreased the Intra-oocyte ROS Level

**Figure 3:**
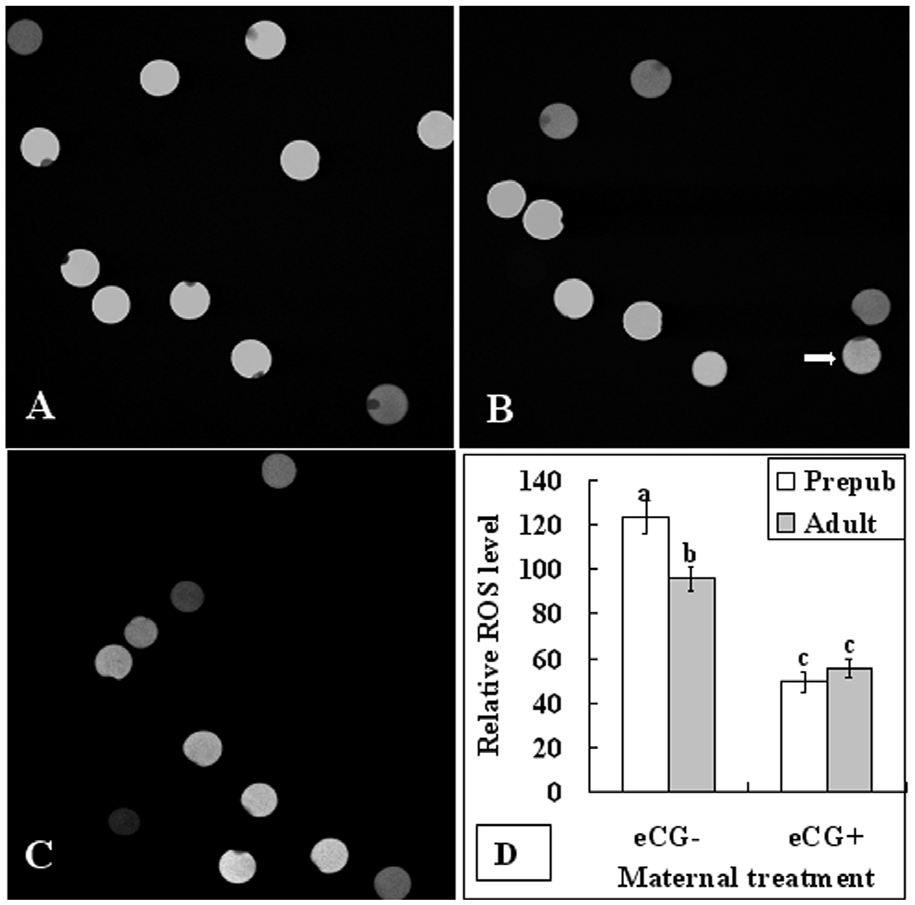
Effects of maternal puberty and eCG treatment on ROS levels of mouse oocytes following in vitro maturation. A to C are confocal images. Images A and B show oocytes from unprimed prepubertal and adult mice, respectively, whereas image C shows oocytes from eCG-primed prepubertal mice. The arrow in image B points to an oocyte with a FIV of 100. Oocytes were observed under a laser confocal microscope following DCHFDA staining. D is a graph showing the relative ROS levels in oocytes from prepubertal (Prepb) or adult mice primed with (eCG+) or without eCG (eCG−). Each treatment was repeated three times with each replicate containing at least 20 oocytes. a–c: Values without a common letter above their bars differ (P<0.05).

In vitro matured oocytes were assayed for intra-oocyte ROS levels at the end of maturation culture. The relative level of ROS in oocytes was determined according to their fluorescence intensity value (FIV), which was calculated by subtracting the background intensity from the total intensity. In unprimed mice, the ROS level was significantly higher in prepubertal than in adult mice (Fig. 3), suggesting that more prepubertal oocytes suffered from oxidative stress. Treatment with eCG significantly decreased the level of ROS in both prepubertal and adult mice and eliminated the age difference.

### Maternal Sexual Maturation, eCG Priming and/or Cysteamine/cystine (C/C) Supplementation to Maturation Medium Improved Blastocyst Formation of Mouse Oocytes

**Figure 4:**
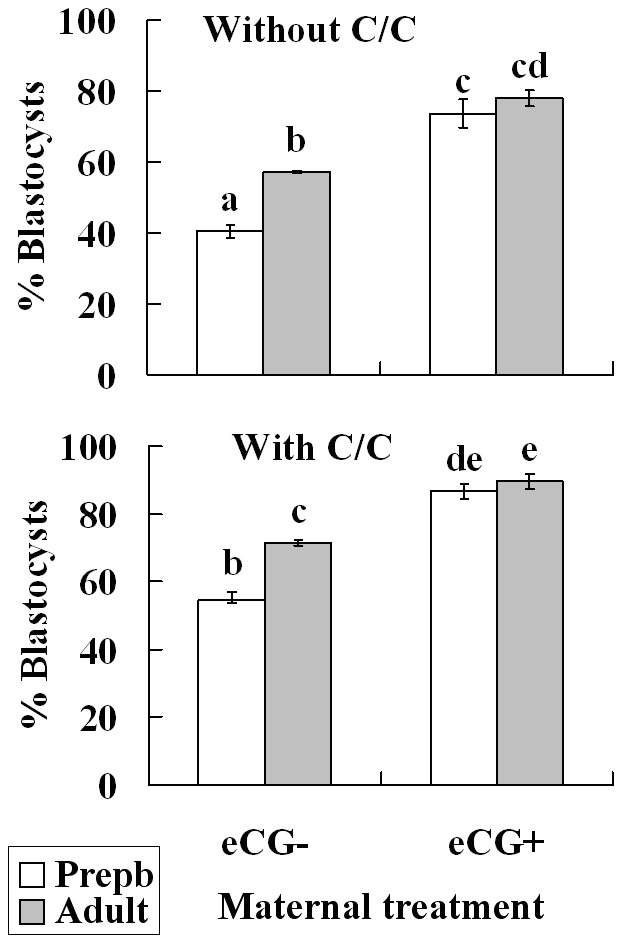
Effects of maternal puberty and eCG treatment and thiol supplementation to maturation medium on blastocyst formation of mouse oocytes. Oocytes from prepubertal (Prepb) or adult mice, primed with (eCG+) or without eCG (eCG−), were matured with or without C (cysteamine, 200 µM)/C (cystine, 400 µM) supplementation, and mature oocytes were inseminated in vitro. Normally fertilized 2PN zygotes were selected and cultured for blastocyst development after insemination. Each treatment was repeated 3–4 times with each replicate containing 15–20 2PN zygotes. a–e: Values without a common letter above their bars differ significantly (P<0.05).

Oocytes from prepubertal and adult mice, primed with or without eCG, were matured with or without C/C supplementation. Maturation rates of oocytes were high (92%–98%) and did not differ between different treatments. Mature oocytes were inseminated and the normal fertilized zygotes with two PN were selected for embryo culture. In unprimed mice, prepubertal zygotes were less capable of blastocyst formation than adult zygotes with or without C/C supplementation (Fig. 4). Maternal eCG priming increased blastocysts of both prepubertal and adult zygotes significantly and to the same level with or without. Thus, C/C supplementation improved blastocyst formation of both prepubertal and adult oocytes, whether primed or not with eCG, but maternal age difference remained in the unprimed oocytes.

### Maternal Sexual Maturation, eCG Priming and/or C/C Supplementation Increased Oocyte Glutathione Synthesis and the GSH/GSSG Ratio

**Figure 5:**
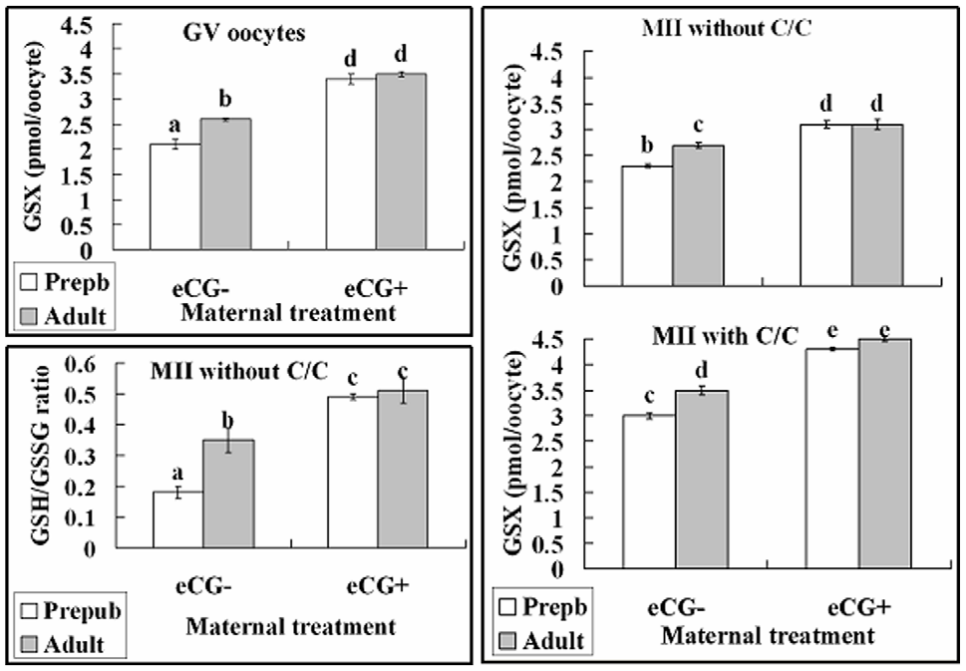
Intracellular GSX concentrations and GSH/GSSG ratio in mouse oocytes. The GSX in oocytes at the GV stage (the upper left panel) were measured immediately after oocyte recovery from unstimulated (eCG−) or eCG-primed (eCG+) prepubertal (Prepb) or adult mice. The GSX concentration (the right panel) and GSH/GSSG ratio (the lower left panel) in MII oocytes were observed after in vitro maturation with or without C (cysteamine, 200 µM)/C (cystine, 400 µM) supplementation. Each treatment was repeated 3 times with each replicate containing 35–40 oocytes. a–e: Values without a common letter above their bars differ (P<0.05) within panels.

Some GV oocytes were assayed for intra-oocyte GSX immediately after collection, whereas others were matured with or without C/C supplementation before assay for GSX and the GSH/GSSG ratio. In unstimulated mice, intra-oocyte GSX was significantly higher in adult than in prepubertal oocytes at both the GV and MII stage (Fig. 5). When mice were primed with eCG, however, the GSX level of both prepubertal and adult oocytes increased significantly and to the same level. C/C supplementation increased GSX synthesis in both eCG-stimulated and unstimulated oocytes but it could not eliminate the age difference in unstimulated mice. The level of GSH was lower significantly in prepubertal than in adult oocytes (0.36±0.04 vs. 0.69±0.07, P<0.05) while the GSSG content was similar between the two age groups (1.97±0.02 vs. 1.96±0.03, P>0.05). As a result, the GSH/GSSG ratio was significantly higher in adult than prepubertal oocytes when mice were not primed with eCG (Fig. 5). However, eCG eliminated the age difference in the GSH/GSSG ratio.

### Maternal Sexual Maturation and eCG Priming Upregulated the Expression of GCL Subunits

**Figure 6:**
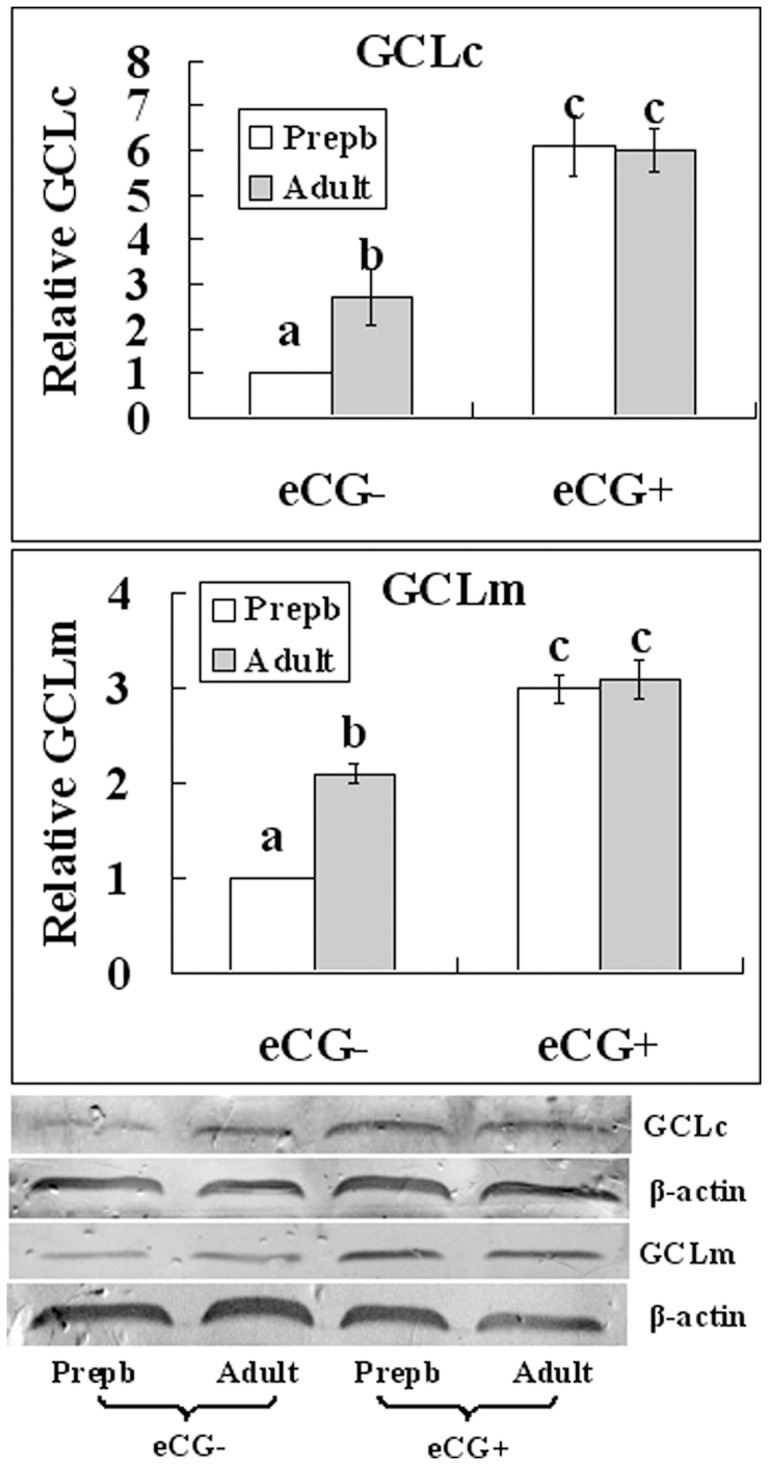
Relative levels of catalytic subunit (GCLc) and modifier subunit (GCLm) of the γ-glutamylcysteine ligase in mouse GV-stage oocytes recovered from unstimulated (eCG−) or eCG-primed (eCG+) prepubertal (Prepb) or adult mice. Results from Western blotting. Each treatment was repeated 3 times and each replicate contained about 500 oocytes. a–c: Values without a common letter above their bars differ (P<0.05) within panels.

Oocytes at the GV stage were processed for Western blot analysis to measure protein contents of the GCL subunits. In unstimulated mice, contents of both GCLc and GCLm were significantly lower in prepubertal than in adult oocytes; but their contents increased significantly and the age difference became insignificant after eCG priming (Fig. 6).

## Discussion

The present study showed that in unstimulated mice, blastocyst rates of adult oocytes were significantly higher than that of prepubertal oocytes, and maternal priming with eCG increased blastocyst rates of both prepubertal and adult oocytes to the same level (Fig. 4). It was reported in various species that the developmental competence of embryos produced from prepubertal animals was lower than that of embryos produced from adult animals [2], [3], [6]–[13] and that priming prepubertal animals with gonadotropin improved their oocyte quality significantly [42], [43]. Increasing evidence demonstrates that oocyte quality depends on the events before GV breakdown, i.e. the oocyte must accumulate the appropriate information for meiotic resumption, fertilization and embryo development before chromosome condensation [44]. However, the events important for ooplasmic maturation that occur during meiotic arrest remain to be specified.

The present results demonstrated that in unstimulated mice, the intra-oocyte GSX concentration was significantly lower in prepubertal than in adult oocytes whether before or after in vitro maturation with or without C/C supplementation (Fig. 5). Previous studies have shown that cysteamine could reduce cystine to cysteine in cell-free medium, promoting cysteine uptake and glutathione synthesis in somatic cells [45] and cumulus-free oocytes [27]. This means that given the availability of cysteine, prepubertal oocytes are still less capable of glutathione synthesis compared to adult oocytes. However, following maternal eCG priming, glutathione of prepubertal oocytes increased to the same level observed in adult oocytes (Fig. 5). Our Western blot analysis showed that protein contents of GCLc and GCLm changed in close consistency with changes observed for intra-oocyte GSX levels (Fig. 6). Thus, the lower glutathione synthesis capacity observed in prepubertal oocytes was a result of their down-regulated expression of GCL, and maternal eCG priming increased glutathione synthesis by up-regulating GCL expression. It was reported in rat that gonadotropins regulated glutathione synthesis of ovarian cells by modulating GCL subunit expression [32], [33], but they did not observe what happened in the oocyte.

This study showed that whereas the GSH/GSSG ratio was lower (Fig. 5), the level of ROS was higher (Fig. 3) significantly in prepubertal than in adult mouse oocytes. It should be noted that the decrease in GSH/GSSG ratio in the prepubertal oocytes was due to the decreased production of GSH because the level of GSH was lower significantly in prepubertal than in adult oocytes while the GSSG content was similar between the two age groups. It is known that once synthesized, glutathione cycles between the GSH and the GSSG form through the actions of glutathione reductase and peroxidase. The peroxidase transfers electrons from GSH to oxidized molecules within the cytoplasm, minimizing the actions of various oxidative stressors and resulting in the production of GSSG [46]. The cellular pool of GSH is maintained by reduction of GSSG to GSH by glutathione reductase as well as further GSH synthesis. Taken together, the present data suggested that oxidative stress had occurred in the prepubertal oocytes due to an impaired GSH synthesis.

In this study, the percentages of oocytes that were not activated at all after sperm penetration were much higher in prepubertal than in adult oocytes in unstimulated mice (Table 1). Our ethanol activation experiment further confirmed the lower ability of prepubertal oocytes to initiate activation. Thus, whereas 84% of adult oocytes were activated, only 63% of prepubertal oocytes were activated after ethanol stimulation. It is well known that sperm activate oocytes by inducing a series of Ca^2+^ spikes. The Ca^2+^ signal activates the anaphase-promoting complexes (APC), leading to the destruction of key proteins necessary for meiotic arrest [47]. Calmodulin-dependent protein kinase II (CaMKII) was found to trigger cell-cycle resumption in mouse eggs and to act downstream of sperm-induced Ca^2+^ release [48]. Thus, the present results that the Ca^2+^ reserves were decreased significantly in the prepubertal oocytes (Fig. 2) confirmed that the poor response of prepubertal oocytes to activation by fertilizing sperm had resulted from their impaired Ca^2+^ releasing capability. It was reported that the regulation of endoplasmic reticulum Ca^2+^ and inositol 1,4,5-trisphosphate receptor (IP_3_R) phosphorylation during maturation enhance IP_3_R sensitivity rendering mouse oocytes competent to initiate oscillations at fertilization [49]. In cattle, although the Ca^2+^ content of intracellular stores was similar, the relative amount and sensitivity of the IP_3_R was significantly lower in calf than in cow oocytes [9], [50].

Because it has been reported that ROS significantly perturb Ca^2+^ homeostasis mainly through direct effects on the machinery involved in intracellular Ca^2+^ storage [38], [39], and that the intracellular Ca^2+^ store was decreased significantly in the in vitro-aged oocytes that may have been exposed to oxidative stress for a prolonged time [40], the present data suggested that oxidative stress had decreased Ca^2+^ reserves leading to impaired activation of prepubertal oocytes.

The present results indicated that percentages of oocytes that failed to form male PN following sperm penetration were significantly higher in prepubertal than in adult mice when not primed with eCG (Table 1). It is well documented that the intra-oocyte glutathione level is important for decondensation of sperm nuclei and the formation of male PN [23], [25], [51]. It is believed that reduction of –S-S- bonds in nuclear protamines and the replacement of protamines by histones are directly involved in sperm nucleus decondensation in the ooplasm [52], [53]. There seems no doubt that the –S-S- reduction of protamines is accomplished by reduced glutathione [23], [54].

This study showed that maternal sexual maturation, eCG priming and C/C supplementation all significantly improved development of selected 2PN zygotes (Fig. 4). This suggested that prepubertal oocytes were still less competent than adult oocytes even if the factors of activation, PN formation and polyspermy were excluded and that their low developmental potential was related to impaired glutathione synthesis. Parthenotes also showed maternal age difference in developmental potential with those from prepubertal oocytes were less capable of blastocyst formation (data not shown). Our previous studies also demonstrated that an increase in intra-oocyte glutathione by C/C supplementation to maturation medium significantly increased blastocyst rates of both goat and mouse parthenotes [26], [27]. It has been reported that cleavage stage embryos have a limited capacity to synthesize glutathione, susceptible to adverse effects of toxicants or conditions that deplete glutathione, but they have the capacity to reduce GSSG [55], [56].

In the present study, percentages of both polyspermic oocytes (Table 1) and oocytes with incomplete CGs migration (Fig. 1) were significantly higher in prepubertal than in adult mice when not primed with eCG. However, the age difference became insignificant after eCG priming. It was also reported in goats [57], sheep [12] and pigs [58] that polyspermy was more frequent in prepubertal than in adult oocytes. An increase of gonadotropin concentration in the culture medium has been found to result in an increase in CGs migration [59]. Furthermore, an elevation in glutathione levels induced by 2-mercaptoethanol and cysteine supplementation during in vitro maturation significantly reduced rates of polyspermic oocytes [60]. However, the mechanisms by which sexual maturity or gonadotropin inhibit polyspermy are not fully understood. The present results showed that ROS increased significantly in prepubertal oocytes due to impaired GSH synthesis, but eCG priming conquered the ROS increase by increasing the GSH level (Figs. 3 and 5). It is known that peripheral CGs migration during oocyte maturation is driven by the microfilaments [36], and the microfilament network was one of the earliest targets of oxidative stress [37]. Thus, oxidative stress induced polyspermy in prepubertal mouse oocytes by damaging microfilaments and affecting CGs redistribution and exocytosis. In addition, oxidative stress may also affect CGs exocytosis directly by perturbing Ca^2+^ oscillations [61].

**Figure 7:**
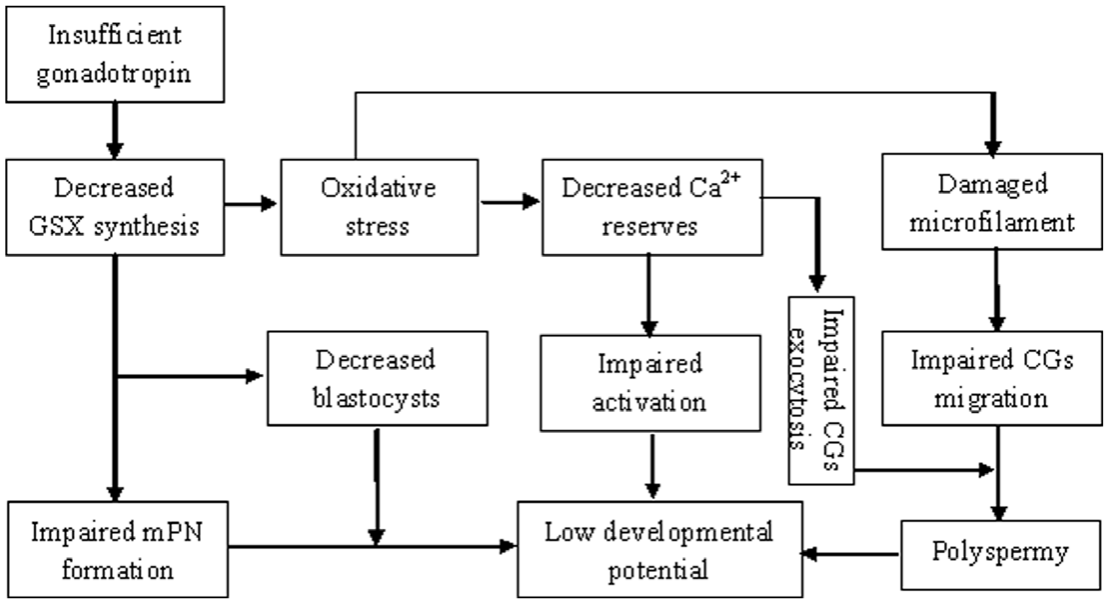
A proposed model showing the possible pathways leading to the low developmental potential in prepubertal mouse oocytes. Refer to text in the last paragraph of the Discussion section for a detailed explanation.

To summarize, a tentative model has been proposed to explain the possible pathways leading to the compromised developmental potential of prepubertal mouse oocytes (Fig. 7). Gonadotropin insufficiency decreases glutathione synthesis by down regulating GCL expression. A decrease in GSH impairs the oocyte’s ability to reduce ROS leading to an oxidative stress. The oxidative stress decreases the Ca^2+^ reserves and affects CGs migration by damaging microfilaments. A decrease in Ca^2+^ reserves impairs oocyte activation and CGs exocytosis. The decrease in GSH also impairs the formation of male PN (mPN) and blastocysts directly. The impaired CGs function results in polyspermy. Impairments in oocyte activation, male PN formation and blastocyst development as well as polyspermy together diminish the developmental potential of prepubertal oocytes.

## Materials and Methods

### Ethics Statement

Mouse care and use were conducted exactly in accordance with the guidelines and approved by the Animal Research Committee of the Shandong Agricultural University, P. R. China (Permit number: 20010510). According to the guidelines of the committee, the animal handling staff (including each post-doc, doctoral or masters student) must be trained before using animals. Mice must be housed in a temperature-controlled room with proper darkness-light cycles, fed with a regular diet, and maintained under the care of the Experimental Animal Center, Shandong Agricultural University College of Animal Science and Vet Medicine. In the present study, mice were sacrificed by cervical dislocation. The only procedure performed on the dead animals was the collection of oocytes from the ovaries.

Unless otherwise specified, all chemicals and reagents used were purchased from Sigma Chemical Co. (St. Louis, MO, USA).

### Animals and Oocyte Recovery

Mice of the Kunming breed were kept in a room with 14−/10-h light-dark cycles, with the dark cycle starting at 8 PM. The prepubertal female mice were used for experiments 21 days after birth, while adult female mice were used 7–9 weeks after birth. Male mice were used for in vitro fertilization 8–12 weeks after birth. Some of the female mice were injected with eCG (10 IU/mouse) at 3 o’clock in the afternoon (prepubertal mice were injected on day 19 after birth) to stimulate follicle development. The eCG-stimulated mice were sacrificed at 46 h after eCG administration, and the unstimulated mice were sacrificed at the same time (unstimulated prepubertal mice were sacrificed on day 21 after birth) to collect ovaries. The large follicles on the ovary were ruptured to release oocytes at the germinal vesicle (GV) stage. Only oocytes >70 mm in diameter with more than three layers of unexpanded cumulus cells and a homogenous cytoplasm were selected for experiments.

### In vitro Maturation of Oocytes

Oocytes were washed three times in M2 medium and once in maturation medium. They were then cultured for 14 h in groups of 20–30 in 100-µl drops of maturation medium at 37.5°C in a humidified atmosphere of 5% CO_2_ in air. The maturation medium was TCM-199 medium (Gibco, Grand Island, NY, USA) supplemented with 10% (v/v) FCS (Gibco), 1 µg/ml 17β-estradiol, 24.2 mg/l sodium pyruvate, 0.05 IU/ml FSH, 0.05 IU/ml LH and 10 ng/ml EGF. Depending on the experiment, different concentrations of cysteamine and cystine were added to the maturation medium. Cysteamine and cystine stocks were prepared at 20 mM and 100 mM, respectively, and diluted to the desired concentrations before use.

### Fertilization in vitro

Masses of dense sperm were collected from the cauda epididymis of fertile male mice and were placed at the bottom of a test tube containing T6 medium supplemented with 10 mg/ml bovine serum albumin (BSA). After 3–5 minutes, the supernatant containing highly motile spermatozoa was removed and capacitated in the same medium u at 37°C for 1.5 h. After being washed in the fertilization medium (T6 containing 20 mg/ml BSA), the oocytes were placed in fertilization drops (around 30 oocytes/100-µl drop). Capacitated sperm were added to the fertilization drops to give a final sperm concentration of about 1×10^6^/ml. The mixed gametes were then incubated for fertilization. Some of the inseminated oocytes were cultured for embryo development at 6 h after insemination, while others were processed for assessment of fertilization at 8 h after insemination.

### Embryo Culture

Inseminated oocytes or selected normal zygotes were washed and cultured for 4 days in the Chatot-Ziomek-Bavister (CZB) medium (around 20 zygotes/100-µl drop) at 37.5°C under humidified atmosphere with 5% CO_2_ in air. Glucose (5.5 mM) was added to CZB when embryos were cultured beyond the 3- or 4-cell stage.

### Assessment of Fertilization

At 8 h after insemination, oocytes were transferred into M2 medium and spermatozoa around the oocyte were removed by pipetting with a small-bore pipette. After washing three times, oocytes were transferred onto a glass slide and covered with a coverslip; petroleum jelly and paraffin wax were used to keep the coverslip in contact with the oocytes without extensive pressure. For fixation, the slides were immersed in ethanol: acetic acid (3∶1, v/v) for at least 24 hours. Fixed oocytes were stained with 1% aceto-orcein and observed by a phase contrast microscope at 400× magnification for PN, sperm heads, sperm tails and/or oocyte chromosomes.

### Cortical Granule (CGs) Staining and Laser Confocal Microscopy

Zona pellucida was removed by treating oocytes with 0.5% pronase (Roche Diagnostics GmbH, Mannheim, Germany; Roche Diagnostic Corp., Indianapolis, IN) in M2 medium. After being washed three times in M2, oocytes were fixed with 3.7% paraformaldehyde in M2 for 30 min at room temperature. The oocytes were then blocked three times for 5 min each in a blocking solution (M2 containing 0.3% BSA and 100 mM glycine). After permeabilization for 5 min in M2 containing 0.1% Triton X-100, oocytes were washed two more times for 5 min each in blocking solution. They were then incubated in 100 µg/ml fluorescein isothiocyanate (FITC)-labeled lens culinaris agglutinin (LCA) in M2 for 30 min in the dark. Finally, the oocytes were washed three times in the washing solution and stained with 10-µg/ml Hoechst 33342 for chromatin examination. After washing, the stained oocytes were mounted on glass slides and observed with a Leica laser scanning confocal microscope (Leica Microsystems GmbH, Wetzlar, Germany). Hoechst 33342-labeled nuclear chromatin was excited with the 405 nm line of a diode laser. The FITC fluorescence was obtained by excitation with a 488 nm line of an Ar/ArHr laser, and the emitted light was passed through a 488 nm filter. The individual optical sections were pseudo-colored and digitally recombined into a single composite image using the Leica Confocal Software.

### Calcium Measurement

Intracellular Ca^2+^ was measured using the Ca^2+^-sensitive dye fluo-3. For loading, oocytes were incubated for 20 min at 37°C with 30 mM of the acetoxymethyl (AM) form of the dye made up in CZB with 0.02% pluronic F-127. Drops of calcium-free M2 were made under paraffin oil in a Fluoro dish (World Precision Instruments, Inc.) with its base coated with phytoagglutinin. After loading, oocytes were washed with calcium-free M2 and placed in the drops and the dish was transferred to a heated stage (37°C) of a Leica laser-scanning confocal microscope (TCS SP2; Leica Microsystems). To measure Ca^2+^ reserves, ionomycin was injected into the drops to produce a final concentration of 2 µM. An argon laser was used for excitation at 488 nm and signals emitted at 505–540 nm were collected for 40 min by the laser scanning confocal imaging system. Traces of calcium oscillations were plotted using Sigma Plot 2000 software.

### Assay for Intracellular Glutathione

Cumulus-free oocytes were washed three times in M2 medium. Five µl of distilled water containing 35–40 oocytes was transferred to a 1.5-ml microfuge tube to which 5 µl of 1.25 M phosphoric acid was then added. Samples were frozen at −80°C and thawed at room temperature. This procedure was repeated three times. The samples were then stored at −80°C until analyzed. Concentrations of total glutathione (GSX) in the oocyte were determined by the 5,5′ dithiobis (2-nitrobenzoic acid) (DTNB)-oxidized glutathione (GSSG) reductase-recycling assay. Briefly, 700 µl of 0.33 mg/ml NADPH in 0.2 M sodium phosphate buffer containing 10 mM ethylenediaminetetraacetic acid (stock buffer, pH 7.2), 100 µl of 6 mM DTNB in the stock buffer, and 190 µl of distilled water were added and mixed in a microfuge tube. Ten microliters of 250 IU/ml glutathione reductase (G-3664) were added with mixing to initiate the reaction. The absorbance was monitored continuously at 412 nm with a spectrophotometer for 3 min, with readings recorded every 0.5 min. To measure the concentrations of GSSG, the samples (10 µl) were vigorously mixed with 0.2 µl of 2-vinylpyridine and 0.6 µl triethanolamine. After 60 min, the sample was assayed as described above in the DTNB-GSSG reductase-recycling assay. Standards (0.01, 0.02, 0.1, 0.2, and 1.0 mM) of GSX and a sample blank lacking GSX was also assayed. The amount of GSX in each sample was divided by the number of oocytes to get the intracellular GSX concentration per oocyte. The reduced glutathione (GSH) values were calculated from the difference between GSX and GSSG for each oocyte and expressed as pmol/oocyte.

### Assay for Intra-oocyte ROS

In order to quantify ROS in individual oocytes, intra-oocyte H_2_O_2_ levels were measured using 2′,7′-dichlorodihydrofluorescein diacetate (DCHFDA) as described by Nasr-Esfahani et al. [41]. Stock solution of DCHFDA was prepared in dimethyl sulfoxide at 1 mM and stored in the dark at −20°C. Immediately before use, the stock solution was diluted to 0.01 mM in M2. Cumulus-free oocytes were stained for 10 min with the DCHFDA solution. After being washed thoroughly to remove the traces of the dye, about 10 oocytes were placed on a slide, covered with a coverslip, and observed under a Leica laser scanning confocal microscope. The fluorescence was obtained by excitation at 488 nm. Photographs were taken using fixed microscopic parameters, and the fluorescence intensity from each oocyte was analyzed using Leica software.

### Western Blot Analysis

Cumulus-free oocytes (n = 500) were placed in a 1.5-ml microfuge tube containing 20 µl sample buffer (20-mM Hepes, 100-mM KCl, 5-mM MgCl_2_, 2-mM DTT, 0.3-mM phenylmethyl sulfonyl fluoride, 3 ug/mLleupetin, PH 7.5) and frozen at −80°C until use. For protein extraction, 5 µl of 5×SDS-PAGE loading buffer was added to each tube, and the tubes were heated (100°C for 5 min). Total proteins were separated on a 12% polyacrylamide gel by SDS-PAGE and transferred electrophoretically on to PVDF membranes. The membranes were washed twice in TBST (150 mM NaCl, 2 mM KCl, 25 mM Tris, 0.05% Tween 20, pH 7.4) and blocked with TBST containing 3% BSA at 4°C overnight. The membranes were then incubated for 2 h at 37°C with mouse anti-GCLc (1∶100, Abcam, Cambridge, MA) or rabbit anti-GCLm monoclonal antibodies (1∶1000, Abcam, Cambridge, MA). After being washed three times in TBST (5 min each), the membranes were incubated for 1 h at 37°C with alkaline phosphatase-conjugated horse anti-mouse IgG (1∶1000, ZSGB-Biotechnology, Beijing, China) for GCLc or alkaline phosphatase-conjugated goat anti-rabbit IgG (1∶2000, Cwbiotech, China) for GCLm. After three washings in TBST, signals were detected by a BCIP/NBT alkaline phosphatase color development kit (Beyotime Institute of Biotechnology, Haimen City, China). Relative quantities of protein were determined with Image-Pro plus software by analyzing the sum density of each protein band image. The quantity values of oocytes from unstimulated prepubertal mice were arbitrarily set as 100% and the other values were expressed relative to this quantity. The quantity of β-actin was used for internal control.

### Data Analysis

There were at least three replicates for each treatment. Percentage data were arc sine transformed and analyzed with ANOVA; a Duncan multiple comparison test was used to locate differences. The software used was Statistics Package for Social Science (SPSS 11.5; SPSS Inc., Chicago, IL, USA). Data were expressed as mean±S.E.M. and P<0.05 was considered significant.
